# Analysis of patient activation and its influencing factors among Chinese rural older adults with chronic multimorbidity

**DOI:** 10.3389/fpubh.2026.1811860

**Published:** 2026-07-21

**Authors:** Xiao Yang, Yanhui Zhang, Danfeng Wang, Xiaofan Wang, Ruofan Qiao, Jixvan Zheng, Chunhui Zhang, Manli Wang

**Affiliations:** 1 School of Nursing and Health, Zhengzhou University, Zhengzhou, China; 2 School of Medicine, Huanghe Science & Technology University, Zhengzhou, China

**Keywords:** chronic multimorbidity, cross-sectional study, older adults, medication burden, patient activation

## Abstract

**Background:**

In recent years, the prevalence of chronic multimorbidity has been increasing year by year. The number of rural patients with chronic multimorbidity cannot be ignored, and such patients are deficient in disease self-management ability. Patient activation plays a positive role in promoting patients to improve self-management behaviors, and is crucial for disease management and prognosis. However, the current status and influencing factors of patient activation among rural patients with chronic multimorbidity remain unclear, which hinders the formulation of targeted and effective interventions.

**Objectives:**

To analyze the current status and influencing factors of patient activation among rural patients with chronic multimorbidity.

**Methods:**

This study adopted a cross-sectional survey design. From January to May 2025, 336 patients aged ≥60 years with chronic multimorbidity residing in Qi County, Kaifeng City, Henan Province, China, were selected as the study subjects. Demographic characteristics, patient activation scores and medication burden scores were collected. Multiple linear regression analysis was performed to explore the influencing factors of patient activation scores.

**Results:**

A total of 336 valid questionnaires were recovered (96%). The mean patient activation score was (46.11 ± 10.93) points. Multiple linear regression analysis showed that age, monthly household income per capita, number of medications, duration of medication use and medication burden were independent influencing factors for patient activation scores among rural older adults patients with chronic multimorbidity (*P* < 0.05).

**Conclusions:**

The overall level of patient activation is low among rural patients with chronic multimorbidity. Targeted attention should be paid to rural patients with chronic multimorbidity who are of older age, have lower monthly household income, take more medications, have longer medication duration, and bear a heavier medication burden. Intensified health education and strong support are warranted to effectively improve their patient activation and ensure quality of life in later life.

## 1 Introduction

Chronic multimorbidity refers to the coexistence of two or more chronic diseases in the same individual. In China, the prevalence of chronic diseases among the older adults is as high as 64.9%, among which the proportion of chronic multimorbidity among rural older adults is 63.3% (1). Studies have demonstrated that people with two existing chronic diseases have a higher risk of developing a third chronic disease (2), and each additional chronic disease increases the risk of mortality by 36% (3). In addition, chronic multimorbidity results in complicated treatment, increased health management costs, and reduced quality of life, posing a major challenge to public health in China.

Patient activation refers to patients' recognition of their own role in self-management, willingness to cooperate with medical staff, possession of knowledge and skills for self-management and health care, and ability to proactively seek health-related information, assistance from medical professionals, and high-quality care (4). Patients with high activation exhibit greater self-management efficacy and are more likely to adopt health-promoting behaviors, thereby achieving favorable health outcomes (5). Multiple studies have indicated (6) that patients with a high level of patient activation are more likely to engage in self-management behaviors, including physical exercise, healthy diet, non-smoking, adherence to medication instructions, and monitoring of their own conditions. These behaviors not only help improve patients' health outcomes but also reduce medical costs.

Medication burden refers to the related burdens experienced by patients during medication use, including medication attitudes, practical difficulties, doctor-patient relationships, therapeutic effects, interference with daily life, side effects, medication behaviors, and economic costs (7). Research has demonstrated that patients' medication burden is a core factor influencing their medication beliefs, medication adherence, and medication safety, as well as an important determinant of their self-medication management and disease outcomes (8). Focusing on patients' medication burden may provide new perspectives for reducing medication-related problems (9, 10). Medication burden affects patients' health behaviors: while individuals recognize the benefits of adopting healthy behaviors, they are also aware of the difficulties associated with changing maladaptive behaviors. The greater the perceived barriers, the lower the likelihood that individuals will adopt healthy behaviors. Conversely, when patients perceive a lower medication burden, they are more likely to value self-management and demonstrate higher levels of patient activation.

For Chinese rural older adults with chronic multimorbidity, a clear research gap exists regarding their patient activation. Relevant evidence (4–6) mostly derives from patients with a single chronic disease or from urban samples, and rarely focuses on this population. Limited by scarce healthcare resources, polypharmacy, cognitive decline, and socioeconomic disadvantages, the patient activation of rural older adults patients with chronic multimorbidity may exhibit unique characteristics, yet the existing literature has rarely investigated this specifically (11, 12).

Based on these considerations, the present study aims to investigate the current status of patient activation levels among rural older adults with chronic multimorbidity and to identify its influencing factors, thereby providing a reference for developing health activation intervention programs targeting this population and further improving public health.

## 2 Methods

### 2.1 Study design

A quantitative, cross-sectional correlational study design was adopted in this study to explore the current status and influencing factors of patient activation among rural patients with chronic multimorbidity. This manuscript was prepared in accordance with the relevant requirements for observational studies stated in the Strengthening the Reporting of Observational Studies in Epidemiology (STROBE) Statement.

### 2.2 Study participants

A mixed sampling STRATEGY combining purposive and convenience sampling was employed. First, for the site selection, a purposive sampling approach based on the principle of typicality was used to choose Qi County, Kaifeng City, Henan Province, China, as the study location. Qi County is situated in the Eastern Henan Plain and is a typical agricultural county. Indicators such as the per capita income of its rural residents, accessibility of medical resources, and the spectrum of major chronic diseases are similar to the average levels in rural areas of Henan Province. Therefore, the county possesses good regional typicality and can effectively represent the agricultural rural areas of the Eastern Henan Plain.

On this basis, from January to May 2025, convenience sampling was conducted. Relying on the chronic disease research collaboration network established by our research team in Qi County, and using multiple township health centers and village clinics in the county as the sampling frame, we consecutively recruited permanent residents with chronic disease multimorbidity who attended consultations or received health management services, ultimately including 336 patients who met the eligibility criteria. The inclusion criteria were as follows: (1) Age ≥60 years; (2) Confirmed diagnosis of ≥2 chronic diseases in a secondary or higher-level hospital; (3) Required to take prescription medications for ≥3 months; (4) Clear consciousness, able to communicate independently with researchers; (5) Voluntary participation in the study. Exclusion criteria were as follows: (1) Complicated with other critical illnesses, such as malignant tumors, severe trauma, etc.; (2) Patients currently participating in other clinical or epidemiological studies.

### 2.3 Research instruments

#### 2.3.1 General data collection

The questionnaire included general demographic information (e.g., age, sex, educational level, marital status.) and disease-related information (disease duration, number of medications).

#### 2.3.2 Patient activation

Patient activation was measured using the Patient Activation Measure (PAM) developed by Hibbard et al. (13) and translated into Chinese by Yang et al. (14) in 2018. The scale consists of 13 items with a 5-level scoring system: scores from 0 to 4 were assigned to responses ranging from “not applicable” to “strongly agree”. The raw sum score was logarithmically converted to a total activation score ranging from 0 to 100, which was further divided into 4 levels. A higher score indicates a higher level of patient self-management activation: Level 1 (low): ≤ 47; Level 2 (low–moderate): 47.1–55.1; Level 3 (moderate): 55.2–67.0; Level 4 (high): ≥ 67.1. The Cronbach's α coefficient of the scale was 0.835. In this study, the Cronbach's α coefficient of this scale was 0.733.

#### 2.3.3 Medication burden

The Medication Lifestyle Questionnaire was developed by the team of Janet Krska (15) and translated into Chinese by Wang (7) in 2020. It is mainly used to assess the medication burden of patients with chronic multimorbidity.The translated questionnaire contains 39 items across 8 dimensions: medication attitude, practical difficulties, doctor–patient relationship, medication effect, interference with daily life, side effects, medication behavior, and economic burden.A 5-point Likert scale was used (1 = “strongly agree” to 5 = “strongly disagree”). The total score was the sum of all items; a higher total score indicates a heavier medication burden. Among them, items 2, 5, 9, 11, 12, 13, 18, 22, 23, 24, 26, 30, 32, 37, and 38 were positively worded items, while the rest were negatively worded items.

The Chinese version of the LMQ has undergone rigorous reliability and validity testing in the target population of this study. The Cronbach's α coefficient for the total scale was 0.855, and the Cronbach's α coefficients for the subscales ranged from 0.822 to 0.932. The intraclass correlation coefficient (ICC) for test-retest reliability ranged from 0.751 to 0.881, all of which met the acceptable criteria (7). Exploratory factor analysis extracted eight common factors, with a cumulative variance explained of 76.780%, which was largely consistent with the structure of the original English version. In the present study, the Cronbach's α coefficient of the scale was 0.810.

### 2.4 Investigation methods

The survey was conducted by four researchers. Before the investigation, all researchers received unified training covering questionnaire distribution, completion procedures, and relevant precautions. Before completing the questionnaire, patients were informed of the purpose, significance, and confidentiality principle of the study, with a promise of anonymity. Questionnaires were distributed after obtaining informed consent and completed by patients independently. For patients unable to finish the questionnaire independently due to visual or educational limitations, researchers read each question aloud and filled in the answers truthfully on their behalf. All questionnaires were completed and collected on-site. Researchers checked the completeness and reminded patients to fill in any missing items. Data entry was performed by two independent researchers to ensure data accuracy.

### 2.5 Statistical methods

A formal *a priori* power analysis was performed using G^*^Power 3.1 software. The test family was set to multiple linear regression (fixed model, R^2^ deviation), with a total of 8 independent variables entered into the model. Following the general conventions in social science research, the significance level (α) was set at 0.05 (two-tailed), the desired statistical power (1–β) at 0.80, and the effect size was specified as medium (f^2^ = 0.15, corresponding to an R^2^ of approximately 0.13). The analysis yielded a minimum required sample size of 109 participants. After accounting for a 10% invalid response rate, the final target sample size was determined to be 121. In practice, a total of 350 questionnaires were distributed, and 336 valid responses were returned (effective response rate: 96%), far exceeding the minimum required sample size. This confirms that the study has sufficient statistical power to detect the prespecified medium effect size.

Data entry and analysis were performed using IBM SPSS Statistics 26.0. Measurement data conforming to a normal distribution were described as (x ± s), and enumeration data were described as frequency and percentage. Univariate analysis was conducted using independent-samples t-test or one-way analysis of variance (ANOVA). Pearson correlation analysis was used to examine the correlation between medication burden and patient activation. Multivariate linear regression analysis was applied for multivariate analysis. A *P*-value < 0.05 was considered statistically significant.

### 2.6 Ethics statement

This study was approved by the Life Science Ethics Committee of Zhengzhou University (Approval No.: ZZUIRB2026-10). Prior to the investigation, all participants were informed of the purpose and procedures of the study. All personal information collected in the study was anonymized and kept strictly confidential. All participants signed a written informed consent form. Participants were also informed that they could withdraw from the study at any time without penalty.

## 3 Results

### 3.1 Baseline characteristics of study participants

A total of 336 rural patients with chronic multimorbidity were included in this study. The age of the participants ranged from 60 to 80 years, with an average of (65.80 ± 3.99) years; among them, 163 were male (48.5%) and 173 were female (51.5%). In terms of marital status, 84 cases (25%) were unmarried, divorced or widowed, and 252 cases (75%) were married. Regarding educational level, 299 cases (89%) had an educational level of primary school or below. For monthly household income, 93 cases (27.7%) had less than 1,000 yuan, 144 cases (42.9%) had 1,000–2,999 yuan, 93 cases (27.7%) had 3,000–4,999 yuan, and 6 cases (1.8%) had 5000 yuan or more. In terms of the number of medications used, 178 cases (53%) took less than 5 kinds, 136 cases (40.5%) took 5–10 kinds, and 22 cases (6.6%) had 11 kinds or more. As for the duration of medication use, 31 cases (9.2%) had a medication duration of less than 1 year, 99 cases (29.5%) had 1–4 years, 151 cases (44.9%) had 5–9 years, and 55 cases (16.4%) had 10 years or more.

### 3.2 Scores of patient activation and medication burden among rural patients with chronic multimorbidity

The average patient activation score of the rural older adults with chronic multimorbidity included in this survey was (46.11 ± 10.93) points, of which 186 cases (55.4%) were at a low level, 72 cases (21.4%) at a low-moderate level, 71 cases (21.1%) at a moderate level, and 7 cases (2.1%) at a high level. The average total score of the medication burden dimension was (109.35 ± 13.27) points, with the average score of medication attitude being (24.93 ± 3.57) points, the score of practical difficulties being (17.06 ± 2.85) points, the average score of the doctor-patient relationship dimension being (10.70 ± 2.53) points, the average score of the medication effect dimension being (10.76 ± 2.65) points, the score of interference with daily life being (15.97±4.49) points, the score of the side effects dimension being (11.57 ± 3.24) points, the score of medication behavior being (9.37 ± 2.46) points, and the economic burden being (9.01 ± 2.45) points.

### 3.3 Correlation analysis between medication burden and patient activation

Correlation analysis showed that patient activation was negatively correlated with total medication burden score (r = −0.278, *P* < 0.05).Specifically, patient activation was negatively correlated with the scores of practical difficulties (r = −0.212, *P* < 0.05), interference with daily life (r = −0.262, *P* < 0.05), side effects (r = −0.280, *P* < 0.05), economic burden (r = −0.177, *P* = 0.001), and medication attitude (r = −0.175, *P* = 0.001).The results are shown in Table 1.

**Table 1:** Correlation analysis between medication burden dimensions and patient activation scores.

Item	Patient activation	Medication burden	Practical difficulties	Doctor–patient relationship	Medication effect	Interference with daily life	Side effects	Medication behavior	Economic burden	Medication attitude
Patient activation	1	–	–	–	–	–	–	–	–	–
Total medication burden	−0.278^*^	1	–	–	–	–	–	–	–	–
Practical difficulties	−0.212^*^	0.729^*^	1	–	–	–	–	–	–	–
Doctor–patient relationship	−0.023	0.434^*^	0.246^*^	1	–	–	–	–	–	–
Medication effect	−0.061	0.492^*^	0.307^*^	0.920^*^	1	–	–	–	–	–
Interference with daily life	−0.262^*^	0.780^*^	0.540^*^	0.099	0.157^*^	1	–	–	–	–
Side effects	−0.280^*^	0.803^*^	0.499^*^	0.104	0.187^*^	0.718^*^	1	–	–	–
Medication behavior	0.089	−0.196^*^	−0.280^*^	−0.028	−0.062	−0.367^*^	−0.277^*^	1	–	–
Economic burden	−0.177^*^	0.633^*^	0.488^*^	0.054	0.086	0.513^*^	0.494^*^	−0.287^*^	1	–
Medication attiude	−0.175^*^	0.488^*^	0.268^*^	−0.193^*^	−0.181^*^	0.301^*^	0.450^*^	−0.177^*^	0.312^*^	1

^*^indicates *P* < 0.05.

### 3.4 Univariate analysis of influencing factors for patient activation among rural patients with chronic multimorbidity

Univariate analysis showed that there were statistically significant differences in patient activation scores among rural patients with chronic multimorbidity with different age, sex, educational level, monthly household income per capita, number of medications and duration of medication use (*P* < 0.05). The results are shown in Table 2.

**Table 2:** Univariate analysis of activation in rural patients with chronic multimorbidity (*n* = 336).

Item	*n*	Score (mean ±SD)	Test statistic	*P*-value
Age	336	46.11 ± 10.93	−0.270^c^	< 0.001
Sex			2.152^a^	0.032
Male	163	47.43 ± 10.87		
Female	173	44.88 ± 10.86		
Educational level			16.160^b^	< 0.001
Primary school or below	299	45.00 ± 10.44		
Junior high school	35	54.67 ± 10.80		
Senior high school or above	2	63.46 ± 5.44		
Marital status			1.216^b^	0.298
Married	252	46.03 ± 10.99		
Unmarried	26	51.85 ± 9.89		
Divorced or widowed	58	45.08 ± 10.81		
Monthly household income per capita			18.364^b^	< 0.001
< 1000	93	43.16 ± 10.23		
1,000–2,999	144	43.80 ± 10.11		
3,000–4,999	93	51.76 ± 10.39		
≥5,000	6	59.94 ± 4.46		
Number of medications			6.278^b^	0.002
< 5	178	47.82 ± 11.08		
5–10	136	44.80 ± 9.74		
≥11	22	40.47 ± 13.76		
Duration of medication use			8.151^b^	< 0.001
< 1 year	31	42.70 ± 9.62		
1–4 years	99	43.92 ± 10.19		
5–9 years	151	46.11 ± 11.09		
≥10 years	55	51.99 ± 10.40		

(1) ^a^*t*-value (independent-samples *t*-test). (2) ^b^F-value (one-way ANOVA). (3) ^c^Pearson correlation coefficient.

### 3.5 Multiple linear regression analysis of influencing factors for patient activation score among rural patients with chronic multimorbidity

The patient activation score was used as the dependent variable. Variables that were significant in the univariate analysis were entered into a multiple linear regression model. The variables were entered as follows: age was entered as a continuous variable; sex was entered as a binary variable (male = 1, female = 2); education level was entered as dummy variables with “primary school or below” as the reference group; per capita monthly household income was entered as dummy variables with “1,000–2,999 ” as the reference group; number of medications was entered as dummy variables with “ < 5” as the reference group; duration of medication use was entered as dummy variables with “5–9 years” as the reference group; and medication burden score was entered as a continuous variable.

The results of multiple linear regression showed that age, monthly household income per capita, number of medications, duration of medication use, and medication burden score were independent influencing factors of patient activation (*P* < 0.05; see Table 3). Sex and educational level were not significantly associated with patient activation in the final model.

**Table 3:** Multiple linear regression analysis of patient activation among rural patients with multiple chronic multimorbidity (*n* = 336).

Variable	B	SE	β	*t*	*p*	95% CI	VIF
(Constant)	83.958	9.455	–	8.880	< 0.001	65.357, 102.559	–
Sex (female vs. male)	−0.804	1.051	−0.037	−0.765	0.445	−2.871, 1.263	1.061
Age (years)	−0.349	0.136	−0.128	−2.560	0.011	−0.617, −0.081	1.158
Medication burden score	−0.142	0.041	−0.172	−3.445	0.001	−0.223, −0.061	1.154
**Educational level (ref: primary school or below)**		
Junior high school	2.538	1.878	0.071	1.351	0.178	−1.157, 6.232	1.266
Senior high school or above	11.154	6.760	0.079	1.650	0.100	−2.146, 24.455	1.037
**Monthly household income per capita (ref: 1,000–2,999)**		
≤ 1,000	0.941	1.279	0.039	0.736	0.462	−1.575, 3.457	1.256
3,000–4,999	7.161	1.285	0.294	5.571	< 0.001	4.632, 9.690	1.267
≥5,000	9.735	4.090	0.118	2.380	0.018	1.689, 17.782	1.127
Number of medications (ref: < 5)							
5–10	−2.233	1.092	−0.100	−2.046	0.042	−4.381, −0.086	1.104
≥11	−5.979	2.174	−0.136	−2.751	0.006	−10.256, −1.703	1.110
**Duration of medication use (ref: 5–9 years)**		
< 1 year	−1.190	1.895	−0.032	−0.628	0.530	−4.918, 2.538	1.229
1–4 years	−0.730	1.242	−0.031	−0.588	0.557	−3.174, 1.714	1.215
≥10 years	4.287	1.511	0.145	2.837	0.005	1.315, 7.260	1.154

B, unstandardized coefficient; SE, standard error; β, standardized coefficient; CI, confidence interval; VIF, variance inflation factor. Dependent variable: transformed patient activation score. Model summary: R = 0.542, R^2^ = 0.294, adjusted R^2^ = 0.265, F_(13, 322)_ = 10.298, *P* < 0.001.

## 4 Discussion

### 4.1 Patient activation of rural patients with chronic multimorbidity is at a low level

In this study, the mean patient activation score was (46.11 ± 10.93), which was similar to the findings of Zhu Yaru et al. (16). The reasons for this phenomenon are as follows: rural patients have not yet completely broken away from traditional medical concepts, and there are problems of passive medical treatment and avoidance of medical treatment, failing to fully establish a sense of responsibility for active disease management (17). Secondly, the patients in this study had a low educational level, making it difficult for them to easily master disease management knowledge and skills. Moreover, when communicating their conditions with doctors, they often struggled to understand professional terminology, resulting in inadequate reception of disease information, insufficient attention to diagnosis and treatment results, and low confidence in disease management (18, 19). The backwardness of mindsets is the deep-seated root cause. Many rural patients have not yet fully shaken off the traditional medical model of “the doctor is the sole authority, and patients only need to comply passively,” and there is a widespread tendency toward passive or even avoidant healthcare-seeking behavior (20). This mindset directly weakens their sense of ownership as the primary person responsible for their own health management, making it difficult for them to actively participate in the decision-making and implementation of disease management. The core of patient activation lies in an individual's knowledge, skills, and confidence. When patients fundamentally do not believe they should or can manage their health, their activation level naturally struggles to improve. In addition, the study found that health education for rural patients was mostly didactic, and some medical personnel devoted more energy to clinical diagnosis and treatment, failing to assume the due responsibilities of health intervention and health education (21). Effective health education should not be a simple accumulation of information, but rather an empowering process based on patients‘ individual differences, cultural backgrounds, and learning abilities (22). This suggests that medical personnel should pay attention to the needs of rural patients, attach importance to health education for patients, guide patients through flexible, personalized and diversified methods, improve patients' health literacy, transform health knowledge into health actions, and effectively improve the level of patient activation (23).

### 4.2 Analysis of influencing factors for patient activation among rural patients with chronic multimorbidity

#### 4.2.1 Age and monthly household income per capita

This study showed that age is an important factor associated with the patient activation of rural older adults with chronic multimorbidity; with the increase of age, patient activation decreases correspondingly, which is consistent with the research results of Zhou Xinyu et al. (24). The reason for this is that older adults patients with chronic multimorbidity who are older have reduced self-care ability, and they often feel physical fatigue and lack of energy, thereby showing an indirect association with their patient activation.

Patients with high monthly household income had a high level of patient activation, which is consistent with the research of Yadav et al. (25). This is mainly because economic strength was related to the quality of life of patients. Faced with high medical expenses, patients with low monthly household income find it difficult to invest economic resources in health, which may hinder patients from obtaining necessary medical and drug treatment, leading to delayed medical treatment or choice of lower-cost treatment methods (26), and reducing patient enthusiasm (27).

#### 4.2.2 Number of medications and duration of medication use

This study found that the number of medications is an inhibiting factor for low PAM scores; the more types of medications used, the lower the PAM score. In this study, 47% of the population took more than 5 types of medications. Studies have shown that when older adults patients take more than 3 types of medications per day, the possibility of non-compliance with medical advice increases, and this possibility increases with the increase in the number and dosage of medications taken (28). In addition, with the increase of age, cognitive level begins to decline, making it impossible for the older adults to remember various medication information, resulting in difficulty in properly storing and keeping medications, difficulty in medication management, reduced willingness to take medications, and loss of confidence in disease management (29), thus their PAM scores are at a low level.

The longer the patient's medication duration, the higher the patient's PAM score. This is mainly because patients who have taken medications for a long time have accumulated a certain amount of experience, have a better understanding of their own preferences and needs, and can be responsible for their own treatment, hence the higher PAM score (30). However, it is important to consider survivorship bias as an alternative explanation for this association. Patients with prolonged medication use represent a selected population that has successfully maintained adherence over an extended period. This group may inherently differ from those with shorter durations, they might have better drug tolerability, more stable disease progression, or stronger initial health literacy and self-management abilities, all of which increase their likelihood of “surviving” in the treatment cohort. Conversely, patients who discontinued medication early due to adverse effects, perceived inefficacy, or rapid disease deterioration are systematically excluded from the long-term user group, and they may also exhibit lower PAM scores. Therefore, the observed positive correlation might partly reflect this differential attrition and selection effect, rather than a pure causal consequence of duration-dependent experience accumulation. Future longitudinal studies are warranted to disentangle the specific contributions of medication experience from the confounding effects of baseline patient characteristics and clinical stability (31).

#### 4.2.3 Medication burden is negatively correlated with patient activation

This study found that higher PAM scores were associated with lower medication burden scores, among which the scores of five dimensions—medication attitude, practical difficulties, interference with daily life, drug side effects, and economic burden—were significantly negatively correlated with the level of patient activation. The most probable explanation is that in the context of multiple morbidities, patients have a low level of understanding of disease knowledge, insufficient awareness of the severity of asymptomatic diseases and insufficient attention to corresponding treatments, insufficient attention to medications (32), failure to develop a strong enough belief in the necessity of medication use (33), and failure to exert the stimulating effect of attitude on behavior, leading to low patient activation scores.

In this study, practical difficulties were mainly reflected in the complex treatment plans for patients with multiple diseases and the weak comprehensive management ability of patients. Furthermore, due to the complex treatment plans, patients had to prioritize medication management to ensure their health, which interfered with their normal social interactions and activities and was not conducive to patients maintaining a high level of activation.

The side effects caused by medications increased patients' discomfort. Studies have found that side effects are an important reason for treatment interruption (34), and during medication use, patients have a fear of drug side effects, especially when they have insufficient information about drug side effects (35), which reduces their confidence in self-management and leads them to take the initiative to give up communicating their conditions with medical staff. A study found that as the number of medications taken increases, patients cannot easily recall the side effects of the medications; however, side effects are moderate, major, or very major problems that are associated with their lives, and they will automatically stop taking medications or adjust medication priorities without prior communication with doctors (36, 37).

Patients have to bear high economic costs for medical treatment. Studies have found that the average two-week expenditure per capita for patients with multiple diseases is 216.05 yuan, while the annual household income is 38,918.28 yuan (38). When patients' health conditions change, they are unwilling to seek medical help in a time.

Our study also found that sex and educational level were significantly associated with patient activation in univariate analyses; however, their statistical significance disappeared after adjusting for confounding factors in the multivariate regression model. This discrepancy may be attributed to the following two aspects. First, confounding effects: univariate analyses failed to control for covariates such as age, household income, and number of medications (39). Second, mediation effects (40): the influence of sex and educational level on patient activation may be partially or fully transmitted through the intermediary variable of “medication burden.” As demonstrated in our study, the total medication burden score remained a consistently significant predictor in the regression model, and its strong explanatory power may have overshadowed the direct effects of demographic variables. These findings suggest that, in clinical interventions, modifiable medication burden, rather than immutable demographic characteristics, should be considered a more critical and direct target for improving patient activation.

## 5 Public health implications

This study, which investigates the current status and influencing factors of patient activation among rural older adults with chronic multimorbidity, provides important public health implications for promoting healthy aging and health equity among the older adults. The findings highlight an urgent need to shift from passive disease treatment to patient-centered proactive chronic multimorbidity management. When implementing health management for rural older adults patients with chronic comorbidities, full attention should be paid to their insufficient health activation. Individualized health guidance and simplified self-management support should be strengthened in primary health care services, and family care and social support systems should be enhanced. Chronic disease follow-up and health education strategies should also be optimized according to their actual needs, so as to improve health management participation and compliance in this population, thereby providing a practical basis for improving the health management model for rural older adults with chronic multimorbidity.

## 6 Limitations

This study has several limitations: (1) The study sample was drawn solely from rural patients in and around Qi County, Kaifeng City, Henan Province, China. Given the convenience sampling at the individual level and the limited sampling scope, caution should be exercised when generalizing the findings to other rural areas. (2) This study adopted a cross-sectional design, which cannot infer the causal relationship between variables. Future studies need to further verify the relevant action paths through longitudinal studies. (3) All measurements in this study used self-reported scales, and the research results may be affected by the subjective judgment bias of the research subjects.

## 7 Conclusion

In this cross-sectional study, patient activation was found to be low, and inadequate disease management cognition and poor medical experience were also prevalent. However, the nature of the association between these two factors and low activation remains to be further examined. It is suggested that healthcare professionals could focus on individualized health education for rural patients with multimorbidity, guiding them to develop a proper understanding of medication side effects and a positive attitude toward disease management. Additionally, patient journey maps could be constructed to provide integrated, whole-disease-course care, with key time points in the healthcare-seeking process marked to reduce waiting and avoid duplicate examinations and treatments. These approaches may potentially help lower medical costs, alleviate economic burden, and contribute to the improvement of medical experience and the enhancement of patient activation.

## Data Availability

The raw data supporting the conclusions of this article will be made available by the authors, without undue reservation.
